# Linear distributed source modeling of local field potentials recorded with intra-cortical electrode arrays

**DOI:** 10.1371/journal.pone.0187490

**Published:** 2017-12-18

**Authors:** Rikkert Hindriks, Joscha Schmiedt, Xerxes D. Arsiwalla, Alina Peter, Paul F. M. J. Verschure, Pascal Fries, Michael C. Schmid, Gustavo Deco

**Affiliations:** 1 Center for Brain and Cognition, Computational Neuroscience Group, Department of Information and Communication Technologies, Universitat Pompeu Fabra, Barcelona, Spain; 2 Ernst StrÜngmann Institute (ESI) for Neuroscience in Cooperation with Max Planck Society, 60528 Frankfurt, Germany; 3 Synthetic Perceptive Emotive and Cognitive Systems (SPECS) Lab, Center of Autonomous Systems and Neurorobotics, Universitat Pompeu Fabra, Barcelona, Spain; 4 Institute of Neuroscience, Newcastle University, Newcastle upon Tyne, United Kingdom; 5 Institucio Catalana de Recerca i Estudis Avancats (ICREA), Universitat Pompeu Fabra (UPF), Barcelona, Spain; 6 Institute for Bioengineering of Catalonia, 08028 Barcelona, Spain; 7 Barcelona Institute of Science and Technology, Barcelona, Spain; Ghent University, BELGIUM

## Abstract

Planar intra-cortical electrode (Utah) arrays provide a unique window into the spatial organization of cortical activity. Reconstruction of the current source density (CSD) underlying such recordings, however, requires “inverting” Poisson’s equation. For inter-laminar recordings, this is commonly done by the CSD method, which consists in taking the second-order spatial derivative of the recorded local field potentials (LFPs). Although the CSD method has been tremendously successful in mapping the current generators underlying inter-laminar LFPs, its application to planar recordings is more challenging. While for inter-laminar recordings the CSD method seems reasonably robust against violations of its assumptions, is it unclear as to what extent this holds for planar recordings. One of the objectives of this study is to characterize the conditions under which the CSD method can be successfully applied to Utah array data. Using forward modeling, we find that for spatially coherent CSDs, the CSD method yields inaccurate reconstructions due to volume-conducted contamination from currents in deeper cortical layers. An alternative approach is to “invert” a constructed forward model. The advantage of this approach is that any *a priori* knowledge about the geometrical and electrical properties of the tissue can be taken into account. Although several inverse methods have been proposed for LFP data, the applicability of existing electroencephalographic (EEG) and magnetoencephalographic (MEG) inverse methods to LFP data is largely unexplored. Another objective of our study therefore, is to assess the applicability of the most commonly used EEG/MEG inverse methods to Utah array data. Our main conclusion is that these inverse methods provide more accurate CSD reconstructions than the CSD method. We illustrate the inverse methods using event-related potentials recorded from primary visual cortex of a macaque monkey during a motion discrimination task.

## Introduction

Multi-electrode recordings of extra-cellular potentials (LFPs) provide a window into the mesoscopic organization of neuronal activity and are a valuable tool in cognitive and perceptual neuroscience [1–7]. Although the physiological content of LFPs is at present not completely understood [8–11], their biophysical origin has been clarified [12]: Extra-cellular potentials reflect volume-conducted transmembrane currents that can be described by a (volume) *current source density* (CSD). Since neural correlates of cognitive and perceptual processes are to be expressed in terms of transmembrane currents, it is of importance to understand the relationship between the dynamics of CSDs and that of the ensuing LFPs in any particular experimental set-up. In particular, the existence of discrepancies between CSDs and LFPs hinder physiological interpretations of experimental results.

For instance, in case of planar recordings of cortical LFPs—which are obtained by inserting a two-dimensional electrode array into cortical tissue at a certain depth—volume-conduction leads to increased propagation speeds and spatial coherence and that these discrepancies between LFP and CSD strongly depend on the inter-laminar organization of the CSD [13]. This study also showed that for oscillatory LFPs, the phases of LFP and CSD might be different, complicating interpretations of spike-field coherence and spike-trigged LFP averages. The nature of LFP-CSD discrepancies can be clarified be considering the formal relation between LFP and CSD, which is described by Maxwell’s equations for electrostatics, that is, Poisson’s equation, which acts as a spatial lowpass filter [12, 14]. Thus, for example, while an evoked potential recorded with a planar array might appear as a single synchronized activation, the underlying CSD might be comprised of a distributed set of local generators. For the above reasons, it is of considerable interest to find out if the transmembrane currents can be reconstructed from the observed extra-cellular potentials.

For inter-laminar recordings of cortical LFPs—which are obtained by inserting a one-dimensional electrode array perpendicularly into the cortex—reconstructing the CSD from observed LFPs is common practice [12, 14] and has yielded valuable information on the laminar organization of evoked, induced, and spontaneous cortical activity [1–3, 15, 16]. The most widely used method to reconstruct inter-laminar CSD profiles from such recordings is the *one-dimensional CSD method* and consists in computing the second-order spatial derivative of the recorded LFPs along the electrode shaft [12, 14]. Although the one-dimensional CSD method is the most straightforward way to reconstruct CSDs underlying inter-laminar LFPs, it presupposes that the tissue conductivity is isotropic and homogeneous and that the CSD is constant in the intra-laminar directions. While cortical tissue is certainly not isotropic and homogeneous [17, 18] and CSDs are constant in the intra-laminar directions, reasonable CSD reconstructions might still be obtained, depending on the experimental set-up. It is, however, difficult to tell *a priori* for any given experimental set-up exactly how critical these assumptions are.

The CSD method can also be applied to planar LFPs by computing the sum of the second-order spatial derivatives of the LFPs along two orthogonal directions of the electrode plane. This *two-dimensional CSD method* has been applied to planar LFPs to investigate the spatial organization of neural activity [19–21]. Besides isotropic and homogeneous conductivity, the two-dimensional CSD method presupposes the CSD to be constant in the inter-laminar direction. This assumption is likely not to be fulfilled as—apart from the finite thickness of the cortical sheet—the inter-laminar profiles of cortical currents typically comprise several sink/source pairs [1–3] and tend to be balanced (but see [22, 23]). As with the one-dimensional CSD method, it is hard to tell *a priori* in which situations the method yields accurate CSD reconstructions. This can be done, however, by using a volume-conduction model of a tissue preparation. Using such simulations, in [13] it was found that the two-dimensional CSD method is reasonably robust to violations of its assumptions, at least when oscillatory phases are concerned. At any rate, it is to be preferred over other reference schemes like the mono-polar (single wire), bi-polar, and average-reference montage. There remains a need, however, for more flexible reconstruction methods, ones that allow incorporating prior knowledge of tissue geometry and conductivity and of the organization of current sources [9, 24].

During the last decade, several reconstruction methods have been developed that use volume-conduction models and thereby allow to explicitly incorporate prior knowledge [25–30]. The general approach is to construct a volume-conduction model of the tissue at hand, that is, a “forward model”, that allows calculating LFPs for any given CSD and subsequently to “invert” the model, that is, to estimate the CSD, given the LFPs. Because generally, the LFP inverse problem is ill-posed in that many different CSDs can account for an observed LFP, uniques of the reconstructed CSD is obtained by exploiting prior knowledge or assumptions on the electrical properties of the tissue and the organization of the CSD. For example, in [25], inter-laminar LFP recordings are inverted by assuming the inter-laminar CSD to be disk-shaped, where the radius of the disk is a free parameter that can be adjusted for a given experimental set-up. In [26], uniqueness is obtained by assuming an inter-laminar CSD profile and using a suitable parameterization of the intra-laminar CSD and by expanding the CSD into an appropriate set of basis functions [27]. In [29], three-dimensional LFP recordings were inverted by minimizing the difference between the observed and predicted CSDs while imposing a penalty on the roughness of the CSD as measured by the norm of its Laplacian. This method thus yields a unique CSD reconstruction by assuming the CSD to be smooth. These and other LFP inverse methods make explicit our assumptions underlying analysis of LFPs and potentially yield more accurate reconstructions than the classical CSD method. As such, they provide a valuable addition to the more traditional tools to analyze extra-cellular potentials [9, 24].

In contrast to the emerging field of LFP inverse modeling [24], inverse modeling of electroencephalographic (EEG) and magnetoencephalographic (MEG) data has a long history and comprises a large body of methods [31]. Although the field of LFP inverse modeling can surely benefit from these methods, it is not immediately clear, however, if they can directly be applied to invert LFPs and how they would perform. An exception is *low resolution electrical tomography* (LORETA) which is a popular EEG/MEG inverse method and has recently been shown to be applicable to three-dimensional LFP recordings [29]. The aim of this study, therefore, is to adapt, test, and apply several EEG/MEG inverse methods on (simulated and experimental) LFP data. Concerning the methods, we focus on the most commonly used distributed inverse methods: the *minimum norm estimate* (MNE), the *weighted minimum norm estimate* (WMNE), *dynamic statistical parametric mapping* (dSPM), *standardized low resolution electromagnetic tomography* (sLORETA), and *low resolution electrical tomography* (LORETA) [31]. When describing these methods, we will point out the commonalities and differences between them and the existing LFP inverse methods [25–30].

## Materials and methods

### A. Forward modeling

#### a1. Continuous LFP forward model

The extra-cellular potential *ϕ* (that is, the LFP) is generated by transmembrane currents that set up an electric field *E* and induce an associated extra-cellular current density *J* in the tissue volume V [12]. The extra-cellular potential is related to *J* by

J=σE=-σ∇ϕ, (1)

where *σ* denotes the conductivity tensor of the tissue [12]. Given that cortical tissue is predominantly organized in the intra- and inter-laminar directions, it is often assumed that *σ* is a diagonal matrix, when expressed in Cartesian coordinates (*x*, *y*, *z*), where (*x*, *y*) denote the intra-laminar (horizontal) and *z* denotes the inter-laminar (vertical) location. Let us denote the diagonal entries of *σ* by (*σ*_*x*_, *σ*_*y*_, *σ*_*z*_). From Eq (1) and using Cartesian coordinates, it follows that

$$\begin{array}{c} (\sigma_{x}\frac{\partial^{2}}{\partial x^{2}}+\sigma_{y}\frac{\partial^{2}}{\partial y^{2}}+\sigma_{z}\frac{\partial^{2}}{\partial z^{2}})\phi =-C, \end{array}$$ (2)

where we have introduced the *current source density* *C*

$$\begin{array}{c} C=(\frac{\partial }{\partial x}+\frac{\partial }{\partial y}+\frac{\partial }{\partial z})J, \end{array}$$ (3)

It is a scalar quantify with the dimension of current per unit-of-volume. Eq (2) is known as the *anisotropic Poisson’s equation*. If the tissue is homogeneous, that is, if *σ* do not depend on location, Eq (2) can be solved by applying the coordinate transformation

$$\begin{array}{c} \left(x^{\prime },y^{\prime },z^{\prime }\right)=\left(\sqrt{\sigma_{y}\sigma_{z}}x,\sqrt{\sigma_{x}\sigma_{z}}y,\sqrt{\sigma_{x}\sigma_{y}}z\right), \end{array}$$ (4)

which converts it into the *isotropic Poisson’s equation*. The latter can be solved explicitly and, after applying the inverse coordinate transformation, yields

$$\begin{array}{c} \phi \left(u,v,w\right)=\int_{V}K_{\sigma }\left(u,v,w,x,y,z\right)C\left(x,y,z\right)dxdydz, \end{array}$$ (5)

where the kernel *K*_*σ*_ is given by

$$\begin{array}{c} K_{\sigma }\left(u,v,w,x,y,z\right)=\frac{1}{4\pi \sqrt{\sigma_{y}\sigma_{z}{\left(x-u\right)}^{2}+\sigma_{x}\sigma_{z}{\left(y-v\right)}^{2}+\sigma_{x}\sigma_{y}{\left(z-w\right)}^{2}}}, \end{array}$$ (6)

[12] and where the integral is taken over the tissue volume V.

#### a2. Discretization of the forward model

For practical use, both sides of the continuous forward model (Eq (5)) need to be discretized. Discretization of the left-hand-side of amounts to calculating the potential *ϕ* at the electrode tips. Discretization of the right-hand-side of such an equation (Fredholm integral equation of the first kind) is generally done either by numerical integration or by expansion of *C* using a set of basis functions [32]. Since *K*_*σ*_ is singular for (*u*, *v*, *w*) = (*x*, *y*, *z*), numerical integration becomes problematic when the electrode tip is located in active tissue (that is, at locations for which *C* ≠ 0). In the case of inter-laminar LFP recordings, the singularity can be dealt with by assuming *C* to have the form *C* = *C*_*h*_*C*_*v*_, where *C*_*h*_ and *C*_*v*_ are intra- and inter-laminar components of *C*, respectively [25, 30]. For a given choice of *C*_*h*_, which corresponds to choosing an *a* *priori* intra-laminar source profile, *C*_*h*_ is integrated out of Eq (5) (either analytically or numerically), yielding a one-dimensional continuous forward model with a non-singular kernel that can be discretized either by numerical integration or basis function expansion. Although in this study we also assume that *C* can be decomposed into an intra- and an inter-laminar component, it might be advantageous for future studies to have a more general way of discretizing Eq (5), which can be done by expanding *C* using suitable basis functions. Before choosing the basis functions, we briefly outline this approach.

The source space of the continuous forward model is the infinite-dimensional vector space of square-integrable functions *C* defined on the tissue volume V, denoted by $L_{2}\left(V\right)$. We now choose *n* linear independent CSDs $\rho_{1},\cdots ,\rho_{n}\in L_{2}\left(V\right)$ and restrict the CSDs to the subspace $H\subseteq L_{2}\left(V\right)$ space spanned by *ρ*_1_, ⋯, *ρ*_*n*_. For every ρ∈H, there are unique coefficients *C*_1_, ⋯, *C*_*n*_ such that

$$\begin{array}{c} C=\sum_{j=1}^{n}C_{j}\rho_{j}, \end{array}$$ (7)

and we can therefore identify every C∈H with the vector of its expansion coefficients *C* = (*C*_1_, ⋯, *C*_*n*_)^*t*^, where *t* denotes matrix transpose. The source space of the discretized forward model hence is the *n*-dimensional Euclidean vector space. By substituting Eq (7) into Eq (5), it follows that

$$\begin{array}{c} \phi \left(x,y,z\right)=\sum_{j=1}^{n}(\int_{V}K_{\sigma }\left(u,v,w,x,y,z\right)\rho_{j}\left(x,y,z\right)dxdydz)C_{j}, \end{array}$$ (8)

which reduces the calculation of *ϕ*(*x*, *y*, *z*) to calculating the potential (at (*x*, *y*, *z*)) generated by the basis functions *ρ*_1_, ⋯, *ρ*_*n*_.

To obtain a discrete forward model, the potential field *ϕ* has to be discretized as well. We thus select *p* locations (*u*_*i*_, *v*_*i*_, *w*_*i*_) (*i* = 1, ⋯ *p*) within the tissue volume V, which correspond to the locations of the *p* electrode tips. Denote the corresponding potentials by Φ_1_, ⋯, Φ_*p*_. Note that this reduces the data space to the *p*-dimensional Euclidean space. Define the *leadfield matrix* $G\in R^{p\times n}$ by

$$\begin{array}{c} G_{i,j}=\int_{V}K_{\sigma }\left(u_{i},v_{i},w_{i},x,y,z\right)\rho_{j}\left(x,y,z\right)dxdydz \end{array}$$ (9)

for *i* = 1, ⋯, *p* and *j* = 1, ⋯, *n*. This gives the discretized forward model

$$\begin{array}{c} \Phi =GC, \end{array}$$ (10)

where Φ = (Φ_1_, ⋯, Φ_*p*_)^*t*^.

Which basis functions are appropriate to represent *C*? In previous LFP inverse modeling studies, different basis functions have been used, including step functions, balls, splines, Gaussians, and data kernels [25–27, 30]. Although choosing the data kernels is an interesting option because it yields a low-dimensional model space, the data kernels of the general forward model (Eq (6)) are singular, which prohibits their use as basis functions in the current context. Instead, as basis functions we choose homogeneous voxels, whose potential has been explicitly calculated for the cubic and isotropic case [33]. In S1 Text, we generalize this formula to the case of rectangular and anisotropic voxels. These are the indicator functions of voxels within the tissue volume V, scaled to have unit norm. The advantage of rectangular monopoles over other basis functions such as Gaussians or balls is that monopolar basis functions are orthonormal, in which case the relationship between the singular value expansion (SVE) of the continuous forward model and the singular value decomposition (SVD) of the discretized model is well-understood [32]. Moreover, their supports constitute a (arbitrary fine-grained) partition of V which allows them to efficiently represent arbitrary current distributions. Another advantage is that only the voxel dimensions have to be chosen, while in the case of balls or Gaussians, besides their dimensions, their locations have to be chosen as well, which introduces unnecessary (free) parameters in the discretization of the forward model.

#### a3. Utah forward model for cortical evoked responses

**Fig 1:**
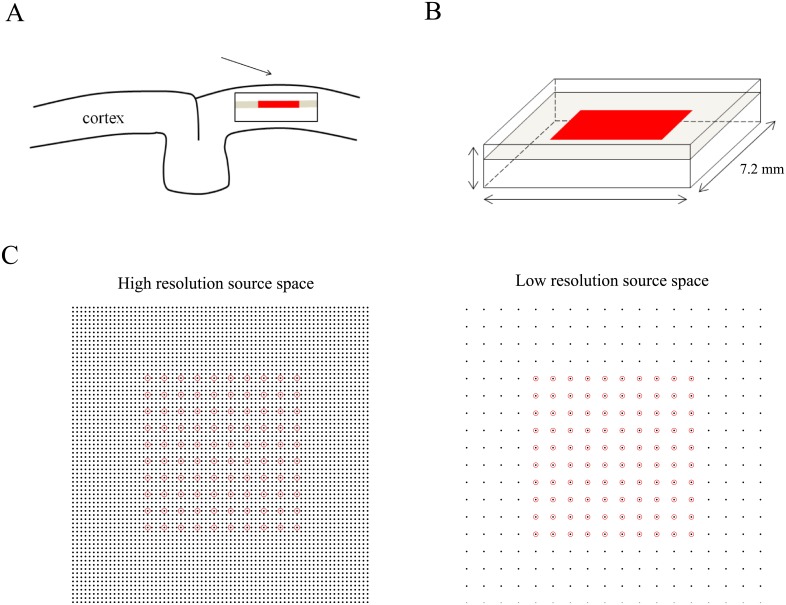
Source space for the Utah array. A. Schematic drawing of a piece of cortex enclosing the rectangular source space (black rectangle) and the Utah electrode array (red line). B. Close-up of the source space and Utah array. The array is located 1 mm under the pial surface. C. Two discretizations of the (intra-laminar) source space (left panel: high-resolution, right panel: low-resolution). Black dots and red circles denote the centers of source voxels and locations of the recording electrodes, respectively. In the high-resolution source space, each cortical slice contains 61 × 61 voxels with intra-laminar lengths of 100 *μ*m. In the low-resolution source space, each cortical slice contains 18 × 18 voxels with intra-laminar lengths of 400 *μ*m. The high and low resolution source spaces comprise 31 cortical slice, each 100 *μ*m thick.

In the simulations we focus on inverse modeling of LFPs recorded with the Utah intracortical electrode array [34], which is one of the most frequently used arrays and comprises 100 electrodes, arranged in a 10 × 10 array with 400 *μ*m inter-electrode spacing. To fully specify the leadfield matrix *G*, a tissue volume V, together with a discrete sampling have to be chosen. We take V to have intra-laminar extent 7.2 × 7.2 mm and inter-laminar extent 3.1 mm, which is about the thickness of the macaque neocortex. Fig 1A and 1B provide illustrations. Note that the intra-laminar extent of V is twice that of the Utah array (which equals 3.6 mm), which allows simulating currents outside the array, which is generally the most realistic scenario. Next, the source space has to be discretized. As the inverse methods behave essentially different for source spaces that have a higher resolution than the Utah array (that is, whose voxel-length is larger than 400 *μ*m) and those that have a lower resolution than the Utah array (that is, whose voxel-length equals 400 *μ*m), we considered two different discretization schemes, corresponding to voxel dimensions of 100 × 100 × 100 *μ*m^3^ and 400 × 400 × 100 *μ*m^3^, respectively. In both cases, the Utah array was placed at a depth of 1 mm at the intra-laminar center of the tissue volume. Fig 1C provides an illustration. The high-resolution source space thus contains *n*_*v*_ = 32 horizontal cortical slices, each containing $n_{h}^{2}=72^{2}=5184$ voxels and the total number of voxels hence equals $n=n_{h}^{2}n_{v}=165888$ [calculate this leadfield again]. The low-resolution source space contains *n*_*v*_ = 31 cortical slices, each containing $n_{h}^{2}=18^{2}=324$ voxels and the total number of voxels hence equals $n=n_{h}^{2}n_{v}=10044$.

An evoked CSD $C\in R^{n_{h}^{2}\times n_{v}}$ is specified by its value on each of the source voxels. It will be convenient to use its *vectorization* $\text{Vec}\left(C\right)\in R^{n\times 1}$ which is obtained by stacking its columns on top of each other. The evoked LFPs $V_{k}\in R^{p\times 1}$ recorded at the *p* = 100 electrode tips of the Utah array at the *k*-trial are given by the following forward model:

$$V_{k}=G\text{Vec}\left(C\right)+\xi_{k},$$ (11)

where $G\in R^{p\times n}$ is the leadfield matrix and where $\xi_{k}\in R^{p\times 1}$ denotes measurement noise which we assume to be normally distributed with expectation zero and covariance matrix $\Sigma_{\xi_{k}}\in R^{p\times p}$ (assumed to be the same for each trial) and to be independent across trials. Note that we assume here that the evoked CSD is the same on every trial. We thus adopt the “signal-plus-noise model” for evoked responses, according to which the CSD can be written as the sum of an evoked response (the “signal”) and a term that models the spontaneous background activity (the “noise”). The signal is assumed to be the same for every trial and the noise is assumed to be not-locked to the stimulus and therefore to be independent over trials. The background activity is absorbed into the measurement noise term. Averaging both sides of Eq (11) over trials yields the trial-averaged forward model

$$\begin{array}{c} V=G\text{Vec}(C)+\xi , \end{array}$$ (12)

where *V* and *ξ* denote the trial-averaged potentials and measurement noise, respectively. Note that the covariance matrix Σ_*ξ*_ of *ξ* equals $\Sigma_{\xi_{k}}$ divided by the number of trials. In the simulations we can assume assume both to be proportional to the identity matrix since the generally large number of trials allows an accurate sample estimate of Σ_*ξ*_, which can subsequently be used to prewhiten the data [32, 35]. Eq (12) describes the trial-averaged forward model whose inversion is the main aim of this study.

### B. Linear distributed source modeling

#### b1. General solution

Inverse modeling aims at “inverting” the forward model for evoked LFPs (Eq (12)), that is, to reconstruct *C* from observed *V*, given the leadfield matrix *G*. In *distributed source modeling*, the reconstructed CSD, denoted by $\hat{C}$, depends linearly on the observed data through an *inverse matrix*, denoted by *G*^♯^:

$$\begin{array}{c} \text{Vec}\left(\hat{C}\right)=G^{♯}V, \end{array}$$ (13)

where *G*^♯^ is given by

$$\begin{array}{c} G^{♯}=\Sigma G^{t}{\left(G\Sigma G^{t}+\Sigma_{\xi }\right)}^{-1}, \end{array}$$ (14)

where Σ_*ξ*_ denotes the trial-averaged noise covariance matrix and $\Sigma \in R^{n\times n}$ can be interpreted as an *a priori* covariance matrix for Vec(*C*) [31]. Indeed, Eq (14) can be derived by following the standard Bayesian procedure using a (multivariate) normally distributed prior on *C*. This class of source modeling methods is called “distributed” because the reconstructions are allowed to be distributed across the source-space, in contrast to EEG/MEG dipole localization methods, in which the number of sources is restricted [31]. Although Eq (14) is the general solution to the inverse problem, in order to obtain reasonable reconstructions, an inter-laminar CSD profile needs to be assumed [26, 27]. To incorporate this assumption into the CSD covariance matrix, we denote the inter- and intra-laminar profiles of *C* by $C_{v}\in R^{n_{v}\times 1}$ and $C_{h}\in R^{n_{h}^{2}\times 1}$, respectively (“*h*” and “*v*” stand for “horizontal” and “vertical”, respectively.). The above assumption means that *C* can be decomposed as

$$\begin{array}{c} C=C_{h}C_{v}^{t}, \end{array}$$ (15)

where *C*_*v*_ assumed to be known. Thus, for each intra-laminar location, the CSD is given by *C*_*v*_ up to a multiplicative constant. Since the vertical CSD profile is assumed to be known, the forward model (Eq (12)) can be reduced to the following *horizontal forward model*:

$$\begin{array}{c} V=G_{h}C_{h}+\xi , \end{array}$$ (16)

where *V* and *ξ* are as in Eq (12) and $G_{h}\in R^{p\times n_{h}^{2}}$ is the horizontal leadfield matrix, which, for each intra-laminar location, is obtained by taking the inner-products of the inter-laminar entries of *G* with *C*_*v*_. The horizontal forward model makes explicit that only *C*_*h*_ needs to be reconstructed. Note, however, that *G*_*h*_ depends on the choice for *C*_*v*_ so that a different choice leads to a different forward model. The solution to the horizontal forward model is given by the following inverse matrix:

$$\begin{array}{c} G_{h}^{♯}=\Sigma_{h}G_{h}^{t}{\left(G_{h}\Sigma_{h}G_{h}^{t}+\Sigma_{\xi }\right)}^{-1}, \end{array}$$ (17)

where $\Sigma_{h}\in R^{n_{h}^{2}\times n_{h}^{2}}$ denotes the *a priori* covariance matrix of *C*_*h*_. The distributed inverse methods that we consider (MNE, WMNE, LORETA, and LORETA*) differ only in the choice of Σ_*h*_.

#### b2. Choices for the *a priori* covariance matrix

The *minimum norm estimate* (MNE) is a popular inverse method within the field of EEG/MEG [31, 36] and corresponds to taking the *a priori* covariance matrix Σ_*h*_ to be proportional to the identity matrix. The *a priori* covariance matrix thus has the following form:

$$\begin{array}{c} \Sigma_{h}^{\text{MNE}}=\sigma_{h}^{2}1_{n_{h}^{2}}, \end{array}$$ (18)

where $\sigma_{h}^{2}$ denotes the *a priori* source variance and $1_{n_{h}^{2}}$ denotes the identity matrix of dimension $n_{h}^{2}$. This means that the MNE assumes the neural currents at two different intra-laminar locations to be uncorrelated and having equal strength. When applied to EEG/MEG recordings, the MNE is known to overestimate superficial sources, for example those located on gyral crowns, and to underestimate deeper sources, for example those located on sulcal walls and fundi. This undesirable property of the MNE is known as *surface bias* [31] because MNE reconstructions tent to concentrate all source power at surface locations in the brain. In Section **b2** of **Materials and Methods** we will see that in the case of two-dimensional LFP recordings, surface bias takes the form of overestimating currents in the proximity of the electrode tips.

One way to reduce surface bias is to counterbalance the bias by weighting the *a priori* source covariance matrix. This gives the *weighted minimum norm estimate* (WMNE) [31]. The WMNE corresponds to the following *a priori* choice for the intra-laminar covariance matrix:

$$\begin{array}{c} \Sigma_{h}^{\text{WMNE}}={\left(W^{t}W\right)}^{-1}. \end{array}$$ (19)

Note that in the special case $W=1_{n_{h}^{2}}$, the WMNE reduces to the MNE. In applications to EEG/MEG data, *W* is usually taken to be a diagonal matrix, thereby reducing the choice of weights to specifying a weighting vector *w* (the diagonal of *W*). Most often the entries of *w* are chosen to be powers of the Euclidean norms of the corresponding leadfields:

$$\begin{array}{c} w_{i}=\left|\right|G_{•,i}{\left|\right|}^{q}, \end{array}$$ (20)

where *G*_•,*i*_ denotes the *i*-th column of *G* (the *i*-th leadfield) and where *q* ≥ 0 is a weighting parameter [36, 37]. Although *q* is usually set to 0.5, its optimal value depends on several factors and determining its optimal value is an empirical issue [37].

Another way of dealing with surface bias is *low resolution electrical tomography* (LORETA), which combines the weighting matrix *W* of WMNE with a constraint on spatial smoothness [31, 38] and has already been successfully applied to three-dimensional LFP recordings [29]. LORETA thus corresponds to the following *a priori* intra-laminar covariance matrix:

$$\begin{array}{c} \Sigma_{h}^{\text{LORETA}}={\left({\left(\Delta W\right)}^{t}\left(\Delta W\right)\right)}^{-1}, \end{array}$$ (21)

where $\Delta \in R^{n_{h}^{2}\times n_{h}^{2}}$ denotes the discrete two-dimensional Laplace operator, which can be written in terms of the (Kronecker) tensor sum ⊕ as

$$\begin{array}{c} \Delta =\Delta_{xx}⊕\Delta_{yy}, \end{array}$$ (22)

where Δ_*xx*_ and Δ_*yy*_ denote the discrete Laplace operators in the *x* and *y* directions, respectively. Since the Laplace operator is a spatial lowpass filter (it performs a local averaging), LORETA biases the reconstructions to spatially smooth ones, which reduces surface bias. *W* we take identical to the weighting matrix defined in the previous section. Because this choice of *W* differs from that in the original version of LORETA [38], we also considered a version of LORETA without weighting, denoted as LORETA*, which corresponds to the following prior structure on the intra-laminar covariance matrix:

$$\Sigma_{h}^{\text{LORETA}*}={\left(\Delta^{t}\Delta \right)}^{-1}.$$ (23)

Table 1 lists the different inverse methods.

**Table 1:** Linear distributed methods considered in this study. First column: abbreviations of the methods’ names: (W)MNE = (weighted) minimum norm estimate, LORETA = low resolution electrical tomography, LORETA* = LORETA without weighting. Second column: corresponding prior structure on the intra-laminar CSD covariance matrix. *W* denotes a weighting matrix and Δ denotes the two-dimensional discrete Laplacian operator.

Inverse method	Prior structure on Σ_*h*_
MNE	$1_{n_{h}^{2}}$
WMNE	(*W*^*t*^ *W*)^−1^
LORETA	((Δ*W*)^*t*^(Δ*W*))^−1^
LORETA*	(Δ^*t*^ Δ)^−1^

#### b3. Model tuning

To actually calculate a CSD reconstruction from observed LFPs, an estimate of the trial-averaged measurement noise covariance matrix Σ_*ξ*_ is required. Because Σ_*ξ*_ is proportional to the single-trial noise covariance matrix $\Sigma_{\xi_{k}}$, it suffices to estimate the latter. This is usually done by averaging the sample covariance matrices of the pre-stimulus data over all trials. Since in event-related studies, there typically are a large number of trials, this estimate will be accurate [35]. The multiplicative factor relating Σ_*ξ*_ to $\Sigma_{\xi_{k}}$ combines with one due to the *a priori* source variances, yielding a free parameter λ > 0 in front of Σ_*ξ*_ in the general solution (Eq (17)), and hence to an inverse matrix *G*^♯^ that depends on λ. To obtain a CSD reconstruction, λ needs to be chosen, a procedure referred to as model tuning. An appropriate value of this *regularization parameter* can be derived from the observed LFPs and a robust and fast method to do this is *generalized cross-validation* (GCV) [39] and has already been shown to work for tuning LORETA reconstructions of three-dimensional CSDs [29]. GCV chooses the value of λ for which the following function *g* is minimized:

$$\begin{array}{c} g\left(\lambda \right)=\frac{\left|\right|\left(GG^{♯}-1_{p}\right)V{\left|\right|}^{2}}{\text{Tr}{\left(1_{p}-GG^{♯}\right)}^{2}}, \end{array}$$ (24)

where 1_*p*_ denotes the *p*-dimensional identity matrix (*p* is the number of electrodes), *V* the observed LFPs, and Tr denotes the matrix trace. GCV is fast and it can be shown to approximate the value of λ obtained by using the computationally expensive leave-one-out cross-validation [39].

We numerically determined the minimum of the function *g* by evaluating it in the values λ = 10^−20^, 10^−19^, ⋯, 10^5^. It is known that GCV generally works best when the measurement noise is uncorrelated, that is (Σ_*ξ*_ = 1_*p*_). In all simulations, we will assume this to be the case and note that the case of correlated noise can be reduced to this case by pre-whitening the data [32]. For evoked responses, this can be done since the large number of trials allow Σ_*ξ*_ to be estimated accurately.

In Section **b2** of **Materials and Methods**, we assess the bias *β* of the difference inverse methods, which, for the general forward model Eq (12), is given by

$$\begin{array}{c} \beta =\left(R_{\lambda }-1_{n}\right)\text{Vec}\left(C\right), \end{array}$$ (25)

where *C* is the true CSD and *R*_λ_ = *G*^♯^*G* denotes the *resolution matrix* associated with the inverse matrix *G*^♯^. Generally, for Tikhonov estimators, ||*β*|| increases as a function of λ, which reflects the trade-off between bias and uncertainty. In particular, in the absence of measurement noise (λ = 0), the bias is minimal, and it is referred to as the *projection bias*. In Section **b2** of **Materials and Methods**, we evaluate the projection bias of the different inverse methods. Due to the finite accuracy of numerically computing the inverse operator, we do not set λ to zero, but to an extremely small value (λ = 10^−30^).

#### b4. Measuring performance

When applying inverse methods to event-related EEG/MEG data, one is often interested in estimating the locations of relatively localized sources. For this reason, the performance of distributed inverse methods is often characterized using resolution matrices [35]. Although resolution matrices contain information on the accuracy of the reconstructions for localized sources, for more extended sources they are less informative [40]. In the case of multi-electrode LFP data, the dipole approximation which is central to inverse modeling of EEG/MEG data is no longer valid and one generally deals with extended source distributions, rather than with discrete point sources. For this reason, we chose to measure the performance of the different inverse methods using the relative mean squared error, which is defined below.

Let $C_{h}\in R^{n_{h}^{2}\times 1}$ be a simulated CSD and let $\hat{C_{h}}\in R^{n_{h}^{2}\times 1}$ be its reconstruction obtained by applying one of the inverse methods to the horizontal forward model (Eq (16)). We compare ${\hat{C}}_{h}$ and *C*_*h*_ by calculating the relative means squared error (rMSE):

$$\begin{array}{c} \text{rMSE}=\frac{\left|\right|C_{h}-{\hat{C}}_{h}{\left|\right|}^{2}}{\left|\right|C_{h}{\left|\right|}^{2}}\times 10^{2}, \end{array}$$ (26)

where the factor 10^2^ is included to express the rMSE as a percentage (instead of a fraction). There are two complications in using this performance measure. First, because *C*_*v*_ will generally not be equal to the true inter-laminar profile, *C*_*h*_ and ${\hat{C}}_{h}$ do not have the same scale and therefore cannot be compared directly. Therefore, before calculating the rMSE, ${\hat{C}}_{h}$ is scaled by a constant *α* so as to minimize $\left|\right|C_{h}-\alpha {\hat{C}}_{h}{\left|\right|}^{2}$, which is achieved for $\alpha =C_{h}^{t}{\hat{C}}_{h}/\left|\right|{\hat{C}}_{h}{\left|\right|}^{2}$. Second, since the horizontal extent of the source space is larger than that of the electrode array, direct calculation of the rMSE will yield relatively high values. This is because the CSD lateral of the electrode array is generally not well reconstructed due to the high dependency of the corresponding leadfields (see S1 Fig). We therefore calculated the rMSE by first restricting *C*_*h*_ and ${\hat{C}}_{h}$ to intra-laminar locations that are covered by the array. The resulting values hence measure the ability of the inverse methods to reconstruct that part of the CSD that is covered by the electrode array. In the rest of the paper, we simply refer to the rMSE as the *(reconstruction) error*.

### C. Evoked responses

#### c1. Simulated data

To test the different inverse methods, we simulate LFPs by applying the trial-averaged forward model to simulated event-related CSDs. Because the CSDs are assumed to be decomposable into an intra- and inter-laminar profile, simulation of an evoked CSD at a given latency amounts to specifying these profiles (*C*_*h*_ and *C*_*v*_). The intra-laminar profiles of experimental event-related responses are diverse and depend on several factors, including the species under consideration, recording region, response latency, state of the preparation, stimulus properties (frequency, duration, strength, etc.), and spontaneous background activity [2, 41–46]. Evoked responses can be confined to single cortical columns (< 500 *μ*m), as is the case for rat barrel cortex after weak stimulation of individual whiskers [43] or be complex and distributed (up to several millimeters in extent) containing depolarizing (source) as well as hyperpolarizing (sink) components, as is the case for odor-induced responses in salamander olfactory bulb [41]. The inter-laminar profiles of evoked responses display similar diversity and stimulus dependencies [1, 14, 47]. Furthermore, while the CSD is commonly assumed to be balanced, recent studies have reported observing unbalanced and monopolar current sources, probably arising through ionic diffusion processes [22, 23].

To be relevant to a broad range of experimental recordings, therefore, we tested the inverse methods on both simple as well as complex and distributed evoked responses. Specifically, the complex-valued intra-laminar current profile *C*_*h*_ is modeled as a superposition of *N* two-dimensional Gaussian densities:

$$\begin{array}{c} C_{h}\left(x,y\right)=\sum_{n=1}^{N}\exp\left(i\phi_{n}\right)\exp(-\frac{{\left(x-x_{n}\right)}^{2}+{\left(y-y_{n}\right)}^{2}}{2\gamma_{h}^{2}}), \end{array}$$ (27)

where (*x*_*n*_, *y*_*n*_) denotes the intra-laminar location of the *n*-th density, *ϕ*_*n*_ denotes a random phase (which gives rise to depolarizing and hyperpolarizing components), and *γ*_*h*_ denotes the spatial width of the densities, which is used to control the spatial scale of *C*_*h*_. We set *N* = 100 and selected the locations randomly from the intra-laminar source space. There is no compelling reason to set *N* = 100 other than that it yields reasonably looking source distributions. The simulation parameters should hence not be taken as claims about the true number of generators of experimental evoked responses; the merely serve to generate a large number of test currents to evaluate the imaging methods. In addition to these responses, we simulated localized responses by setting *N* = 1, *ϕ* = 0, and (*x*_1_, *y*_1_) = (3.4, 3.4) (that is, in the center of the electrode array).

The inter-laminar current profile *C*_*v*_ is modeled as a superposition of two one-dimensional Gaussian densities of equal amplitude and opposite sign and centered at depths *z*_0_ ± *L*/2:

$$\begin{array}{c} C_{v}\left(z\right)=\exp(-\frac{{\left(z-\left(z_{0}+L/2\right)\right)}^{2}}{2\gamma_{v}^{2}})-\exp(-\frac{{\left(z-\left(z_{0}-L/2\right)\right)}^{2}}{2\gamma_{v}^{2}}). \end{array}$$ (28)

This parametrization corresponds to a dipolar profile with poles located at depths *z*_0_ ± *L*/2 thus having length *L*. We set *L* = 0.8 mm and treat *z*_0_ as a free parameter (see below). Furthermore, *γ*_*v*_ models the width of the poles and is kept fixed at *γ*_*v*_ = *L*/3.

The trial-averaged evoked LFPs are subsequently calculated by applying the leadfield matrix (Section **Utah forward model for cortical evoked responses**) and subsequently adding measurement noise. We take the covariance matrix Σ_*ξ*_ of the trial-averaged measurement noise to be a diagonal matrix with variance $\sigma_{\xi }^{2}$. Because the variance of the measurement noise is inversely proportional to the number of trials, which varies from study to study, we consider different noise-levels. Specifically, following [29], we set

$$\begin{array}{c} \sigma_{\xi }^{2}=0.01\beta {\hat{\sigma }}_{V}^{2}, \end{array}$$ (29)

which expresses the variance of the noise as a percentage *β* of the (sample) variance ${\hat{\sigma }}_{V}^{2}$ of the noise-free vector of recorded potentials *V*, where *β* ranges from 1 to 20 in steps of 2.

**Fig 2:**
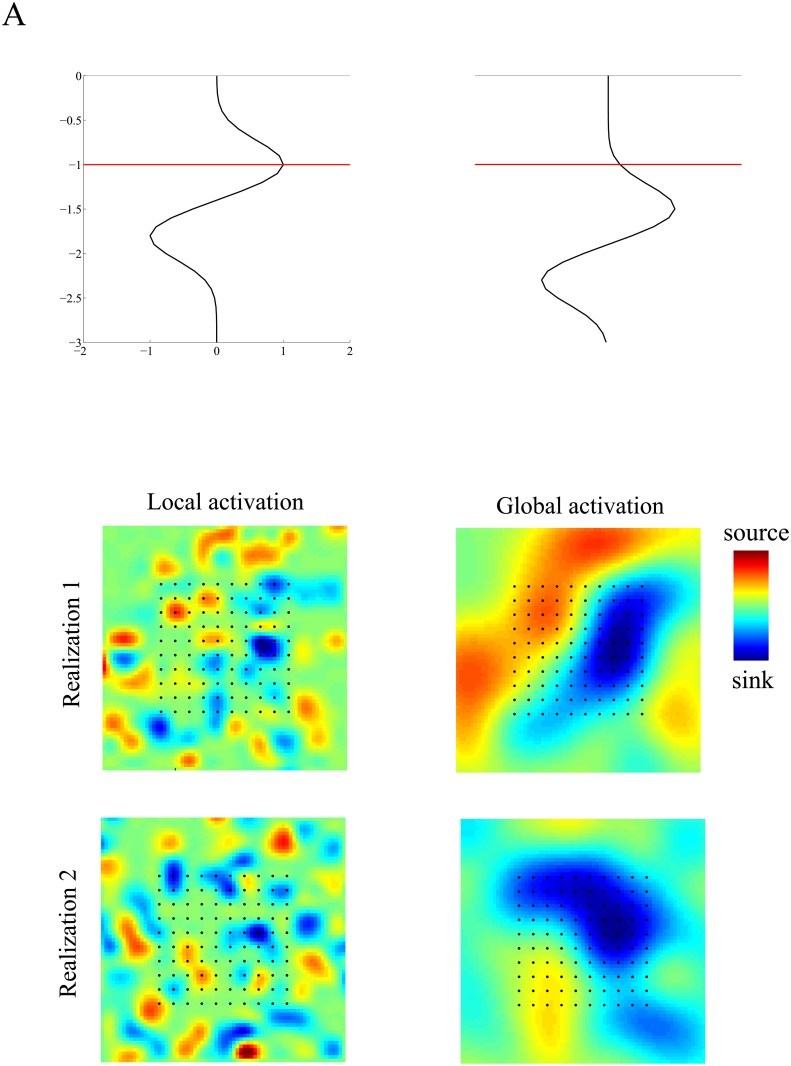
Simulation of evoked current source densities. A. Inter-laminar current source density (CSD) component modeled by superficial (left) or deep (right) dipolar profiles. Deep and superficial are with respect to the electrode array, which is located at a depth of 1 mm (red line). Zero mm corresponds to the pial surface. B. Two (independent) realizations of simulated intra-laminar CSD components corresponding to local (left column) and global (right column) activations. Red and blue correspond to current sources (depolarization) and current sinks (hyperpolarization), respectively. Black dots denote the electrode locations (400 *μ*m inter-electrode spacing).

In all simulations except the high-resolution simulations in Section **b2** of **Materials and Methods**, we vary two key parameters: the width of the intra-laminar current profile (*γ*_*h*_) and the depth of the current generator (*z*_0_). Specifically, each of these two parameters takes on two different values (see Table 2). This means that when assessing the effect of errors in the inter-laminar current profile, four sets of simulations are performed for each of the five noise-levels. For each of the four combinations, the reconstruction errors of every inverse method are averaged over 500 independent realizations. To be able to accurately compare the results obtained for different parameters and different types of errors in the *a priori* inter-laminar current profile, we used the same 500 realizations of all random variables appearing in the simulations (the intra-laminar locations, initial phases, and measurement noise). Fig 2A shows that inter-laminar current profiles of the superficial and deep generators and Fig 2B shows two realizations of their intra-laminar profiles.

**Table 2:** Free parameters and their values. Listed are the free parameters, their symbols, units, and the two values they take on (Value 1 and Value 2).

Parameter	Value 1	Value 2
Intra-laminar width (*γ*_*h*_)	0.2 mm (local)	0.8 mm (global)
Depth (*z*_0_)	1.4 mm (superficial)	1.9 mm (deep)

#### c2. Experimental data

Electrophysiological data were collected from primary visual cortex of a macaque monkey (*Macaca mulatta*) that was performing a motion discrimination task. All experimental procedures were in accordance with the animal welfare guidelines of EU directive 2010/63/EU. Ethical review and permission for this work was granted (F149/05) by the regional board Regierungspräsidium Darmstadt. The monkey was group housed with other macaques in facilities of the Ernst StrÜngmann Institute for Neuroscience in accordance with German and EU regulations. The facility provides an enriched environment including toys, wood, natural daylight access and exceeds the size requirements of EU regulations. The monkey received unrestricted access to food and fluids for the duration of the study. On training and recording days, fluid access was controlled contingent on performance during the task. The monkey was bred and purchased from Health Protection Agency, Salisbury, UK. At the end of the study all implants for electrophysiological data collection were removed and the monkey continued to live in his group.

For array and headpost implantation surgeries, anesthesia was induced with a Ketamin/Dexmeditomidin injection and maintained with volatile Isoflurane. Pain was managed with Remifentanil. The data analyzed here is from the trial period where the monkey only had to keep fixation. Black and white square-wave gratings (80% contrast, horizontal orientation, static, 2° diameter, 1.25 cycles/°, surrounded by a 2 px wide annulus with 2.6° diameter) were presented on a 24” Samsung 2233RZ screen at 120 Hz refresh rate at a viewing distance of 86 cm with gray background. The monkey had to keep fixation within 0.6° radius on a small fixation spot in the center of the screen. Stimuli were presented at 4.5° of eccentricity. Local field potentials (LFPs) were acquired from a 64 multi-electrode grid (“Utah” array, 8 × 8 layout, 400 *μ*m inter-electrode spacing, 1 mm electrode length) using a CerePlex E headstage and connected to a Cerebus System (Blackrock Inc.) at 30 kHz sampling rate. The reference electrode was a small wire (∼ 2 mm) reaching out of the array. For the analysis, 535 number of trials were available.

## Results

### A. Performance of the CSD method

The most critical assumption underlying the two-dimensional CSD methods is that the inter-laminar CSD profile is constant (and of infinite extent). Indeed, when the CSD profile is taken to be constant, the average reconstruction error for the standard set of simulations and in the absence of measurement noise equals 4.6%. This shows that the assumption of an infinite inter-laminar extent is not critical and that a cortical thickness of about 3 mm suffices to obtain accurate CSD reconstructions. Experimental inter-laminar CSD profiles, however, are far from being constant and typically comprise multiple dipolar generators as predicted by standard cable theory (but see [22, 23]). It is therefore unclear to what extent the CSD method can successfully be applied to planar LFP recordings. In [13] it has been shown that, at least theoretically, the reconstructed spatial phase-patterns of oscillatory CSDs resemble the true phase-patterns relatively well and that the CSD method is to be preferred over mono-polar (single-wire) and average-reference montages. In the current study we are not concerned with oscillatory phase-dynamics but with evoked responses and is it *a priori* unclear how accurate the CSD method is in this context. Answering this question will also enable us to assess under which conditions the CSD method is to be preferred over tomographic imaging.

**Fig 3:**
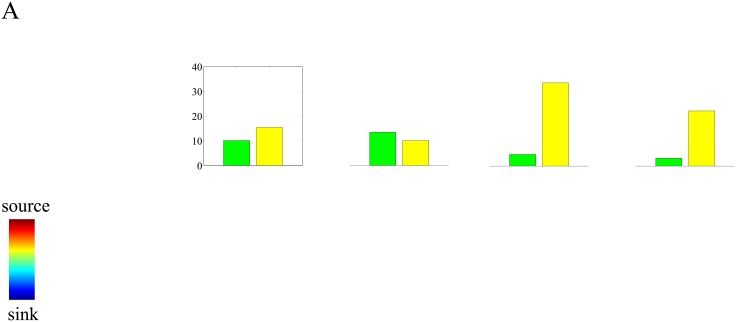
Limitations of the two-dimensional CSD method. Bar plots of the average reconstruction errors for the (numerical) CSD method (yellow) and the MNE inverse method (green) and under each of the four CSD configurations. Errors were obtained by averaging over 500 independent realizations.

To this end, we applied the numerical CSD method to the standard set of simulations and compared it with MNE, since the other imaging methods yielded similar results. Since we are interested in the fundamental limitations of the CSD method, we used it in its most basic form [14] while assuming zero measurement noise. Also, when including the boundary electrodes, the CSD method yields high reconstruction errors, and we therefore excluded these electrodes in the calculation of the reconstruction errors. The results are summarized in Fig 3. The figure shows that for sources with local intra-laminar activations, the errors of the CSD method and the inverse method (MNE) are comparable. For global sources, however, the CSD methods yields much higher errors than MNE. The cause of the poor reconstructions of the CSD method in case of global sources is that spatially coherent currents in layers other than in which the electrode array is located, induce strong electric fields which contaminate the potentials measured at the electrodes [13]. These results demonstrate that the CSD method can be improved upon by using inverse methods, at least in the absence of measurement noise and errors in the forward modeling. At any rate, it provides sufficient motivation for the development and evaluation of such methods.

### B. High-resolution imaging

#### b1. The Utah leadfield matrix

The Utah leadfield matrix *G*—which maps cortical currents to extra-cellular potentials—contains the electrical properties and geometry of the tissue and recording set-up, and as such, sets limits on what can be recovered by the different inverse methods. Therefore, before considering the inverse methods, it will be instructive to have a closer look at the leadfield matrix itself and especially the high-resolution leadfield matrix. Although the *a priori* choice of an inter-laminar current profile reduces the inverse problem to a two-dimensional (intra-laminar) problem by collapsing the inter-laminar dimension of *G*. in this section we consider the full three-dimensional leadfield matrix. For one thing, considering the inter-laminar dimension of *G* aids in understanding how inter-laminar volume-conduction effects “contaminate” cortical LFPs [48]. We analyze *G* through the sensitivities of its constituent leadfields.

**Fig 4:**
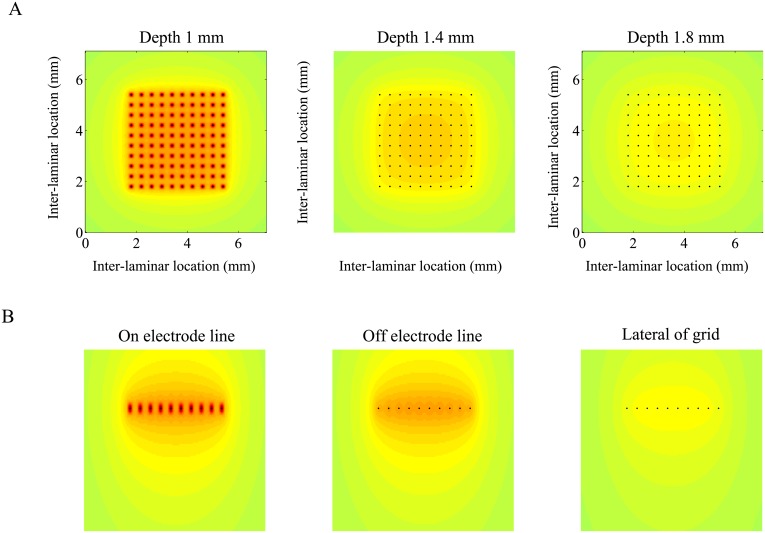
Sensitivity of the Utah leadfield matrix. A. Sensitivity profile of the leadfields along three intra-laminar (horizontal) slices at different cortical depths (left: 1 mm, middle: 1.4 mm, right: 1.8 mm). The Utah array is located at a depth of 1 mm. B. Sensitivity profile of the leadfields along three vertical slices at different lateral locations (left: through the center of the array and on the electrode line, middle: through the center of the array and off the electrode line, right: 200 *μ*m lateral to the array). In all panels, the same color-scaling has been applied so that the sensitivities can be directly compared. Green and red correspond to low and high values, respectively. Black dots in A and B denote the electrodes of the 10 × 10 Utah electrode array (400 *μ*m inter-electrode spacing).

The *sensitivity* of the *k*-th leadfield of *G*, that is, its *k*-th column, is defined to be its Euclidean norm. It is a measure for the strength with which the corresponding monopolar current of unit amplitude contributes to the array potentials. Because the columns of the LFP leadfield matrix are the leadfields of *monopolar* currents, all entries are positive. This is in contrast to MEG/EEG leadfields, which contain the sensor-projections of *dipolar* currents and hence generally contain both positive and negative entries. Fig 4A shows the sensitivity profiles of *G* along three horizontal slices through the modeled tissue volume. The left panel shows the sensitivity profile at the depth of the array (1 mm). It shows that currents at the electrode tips (the black dots) contribute the strongest to the recorded LFPs and that sensitivity drops sharply lateral from the array. The middle and right panels shows that with increasing depth (relative to the electrode array) the sensitivity profile becomes more homogeneous (middle and right panels) and decreases steadily. Fig 4B shows the sensitivity profiles for three vertical slices through the modeled tissue. The fast decrease in sensitivity with depth is clear from the left and middle panels, while the right panel shows that the sensitivity for locations lateral to the electrode array is relatively homogeneous.

Since the sensitivity of the LFP leadfields decreases fast for locations outside the electrode array (in both intra- and inter-laminar directions), it might seem as if volume-conduction does not pose difficulties in the interpretation of LFP data. However, the contribution of a current at a particular location is obtained by weighting the locations’ sensitivity with the strength of the current at that location and this is why LFPs can contain contributions from neural activity several millimeters or even centimeters away. In [1, 48], for example, visually and auditory evoked potentials are shown to contain contributions of different belts of primary sensory cortices. It also explains the often observed discrepancies between simultaneously recorded voltage-sensitive dye (VSD) signals—which are not contaminated by volume-conduction—and LFP recordings from the same region [13, 49].

#### b2. Proximity bias

The MNE inverse method solves a penalized least squares problem in which the penalty is proportional to the power (that is, the squared Euclidean norm) of the current distribution [31]. When applied to EEG and MEG data, this has the undesirable consequence that the power of reconstructed current distributions tends to concentrate in locations that are “electrically close” to the EEG electrodes/MEG sensors. More precisely, current power is over- and under-estimated at locations whose corresponding leadfields have high and low sensitivity, respectively. Since electric and magnetic fields attenuate with distance, EEG and MEG leadfield sensitivities correlate with physical proximity. The consequence is that current power will be overestimated in cortical gyri—which are close to the electrodes/sensors—and underestimated in cortical sulci. For EEG and MEG data, this “surface bias” in MNE reconstructions can be reduced by weighting the leadfield matrix with the inverse leadfield sensitivities (yielding the WMNE), by spatial smoothing (yielding LORETA), or by appropriate normalization of the MNE reconstructions (yielding dSPM and sLORETA) [31].

Although in the case of intra-cortical LFPs there is no surface bias, leadfield sensitivities can still be analyzed and it is important, therefore, to consider the presence of a more general bias, which we will refer to as *proximity bias*. Although the sensitivities of the LFP leadfield matrix are all finite—due to the use of voxels instead of point monopoles—in the previous section we have seen that the leadfields at the electrode locations are particularly sensitive (Fig 4A, left panel). This suggests that MNE reconstructions might be biased towards electrode locations and that one of the other imaging methods might perform better. Fig 4A also showed that proximity bias is largest in the intra-laminar plane containing the electrode array. To assess the presence of a proximity bias, we therefore simulated CSDs confined to a single high-resolution intra-laminar slice containing the electrode array.

**Fig 5:**
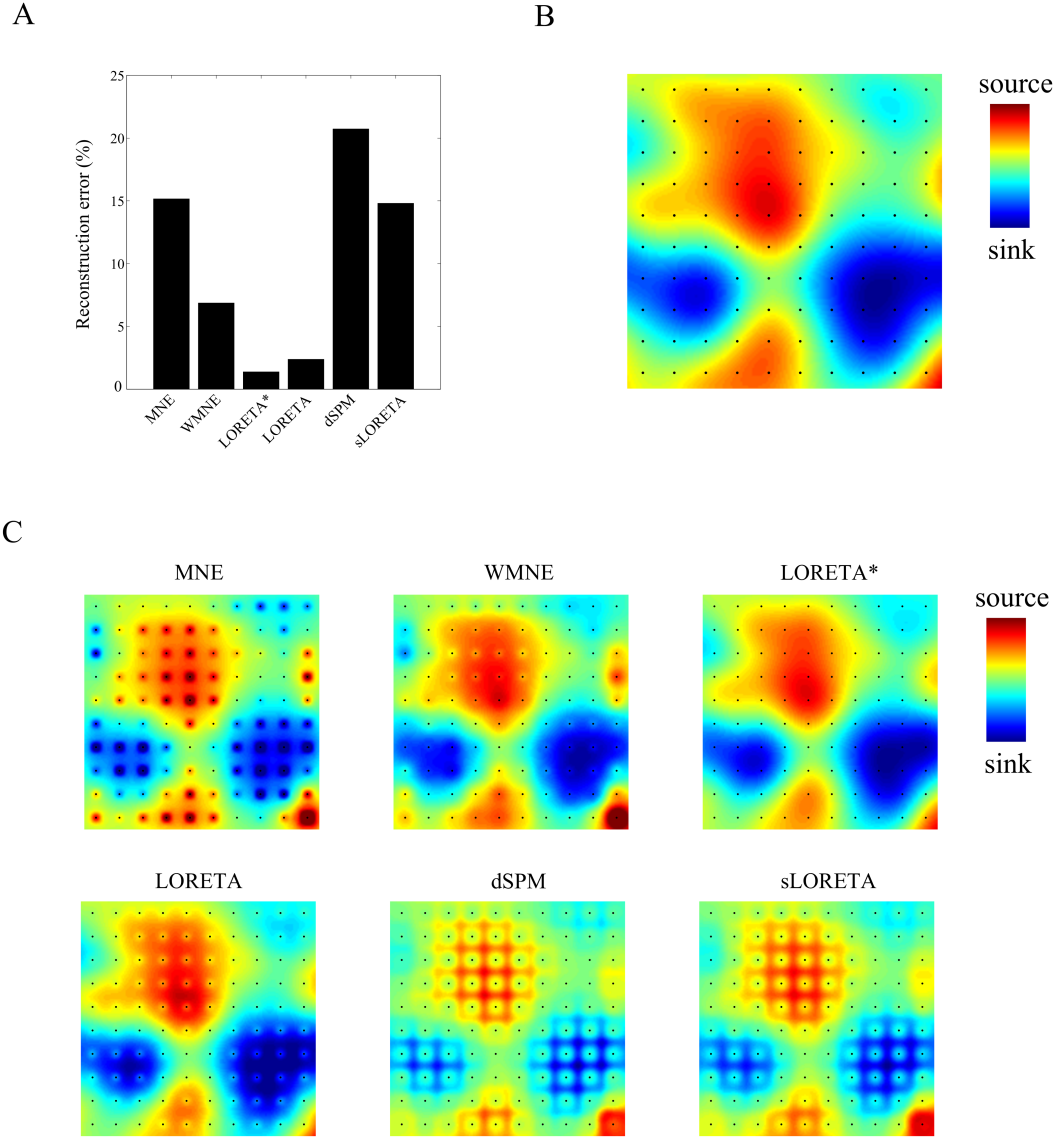
Proximity bias in high-resolution LFP imaging. A. Reconstruction errors averaged over 100 independently generated evoked responses for each of the inverse methods. Intra-laminar spatial width of the responses was set to 0.45 mm, measurement noise was set to zero, and the responses were confined to the intra-laminar slice containing the electrode array. B. Single realization of a simulated evoked response (showing only the part that is covered by the electrode array). C. Reconstructions of the response in B. using the different inverse methods (MNE, WMNE, LORETA*, LORETA, dSPM, and sLORETA).

Fig 5A shows the reconstruction errors averaged over 100 independently generated evoked responses with intra-laminar spatial width equal to 0.45 mm and measurement noise set to zero. Note that MNE has relatively large errors compared to WMNE, LORETA, and LORETA*. These errors are largely due to proximity bias as becomes clear when inspecting the reconstructions (Fig 5C) of a single simulated CSD (Fig 5B). Note that MNE overestimates the currents at the electrodes locations and (slightly) underestimates the currents at locations in between the electrodes. In other words: MNE tends to current power in the electrode locations, that is, it suffers from proximity bias. Although weighting of the leadfields (WMNE) yields smaller errors (Fig 5A), it does not completely remove the bias. Following [37], we have tested a range of values for the weighting parameter *p*, but this did not substantially reduce the bias. Fig 5A also shows, however, that WMNE is more effective in reducing proximity bias than normalization of MNE reconstructions (dSPM and sLORETA), which tends to overcompensate, leading to underestimation of current power at the electrode locations (see Fig 5C). We did not consider alternative normalization matrices and it therefore remains an open question to what extent dSPM and sLORETA can be adjusted to more effectively reduce proximity bias. It seems that LORETA* reconstructions are free of proximity bias, which is due to its spatial smoothing and absence of weighing (as is done in LORETA).

In the remainder of the manuscript, we restrict to imaging using the low-resolution source-space for which proximity bias is absent. We do not further consider dSPM and sLORETA as they practically yielded the same results as MNE.

### C. Low-resolution imaging

#### c1. Performance of the imaging methods

**Fig 6:**
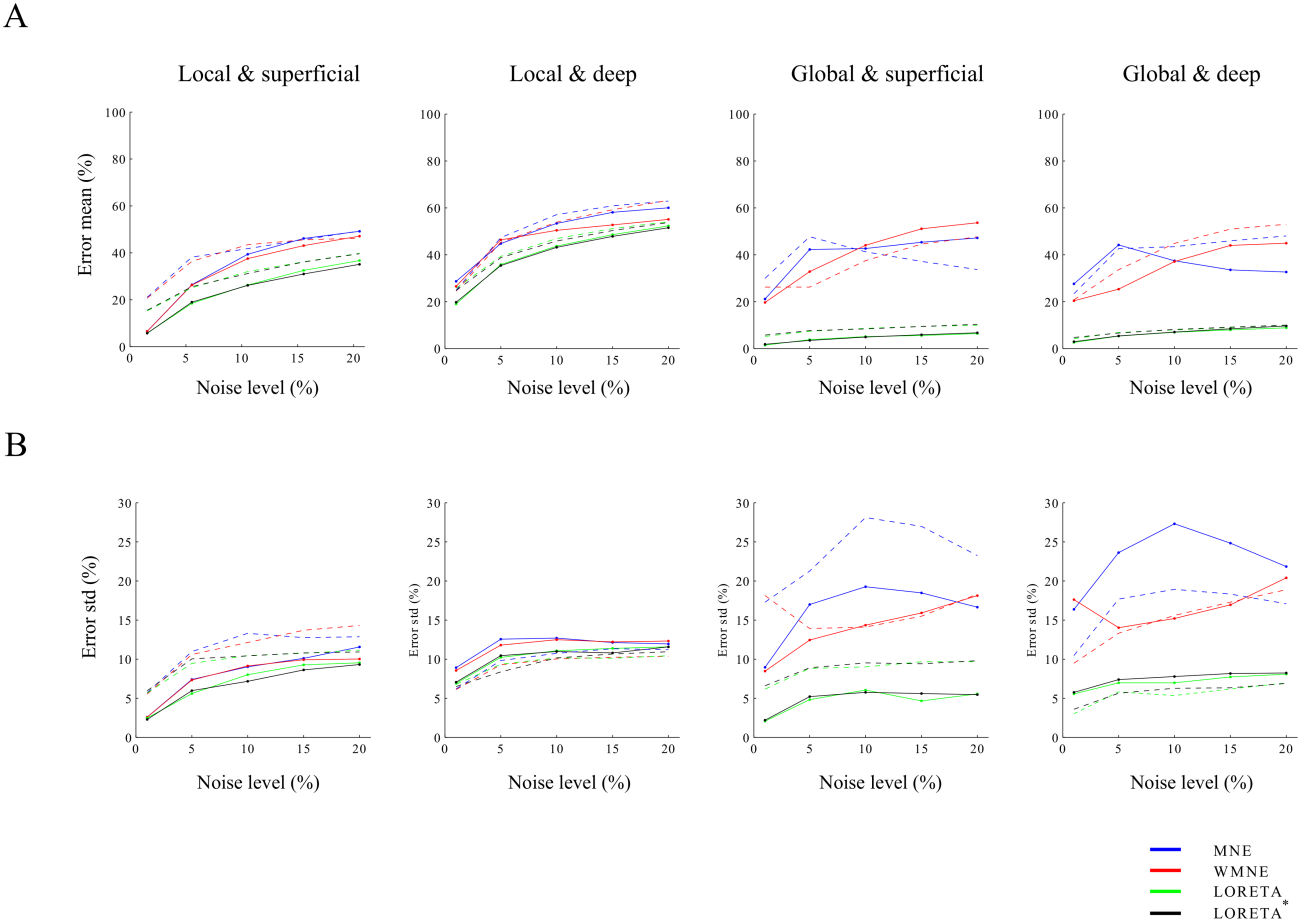
Performance of the imaging methods. A. Mean reconstruction errors for the four inverse methods (MNE (blue), WMNE (red), LORETA (green), and LORETA* (black)) as a function of noise-level and for each of the four combinations of simulated currents (superficial/deep and local/global). Noise-levels are 1, 5, 10, 15, and 20%. The mean errors were obtained by averaging over 500 realizations. B. Same format as in A. but displaying the error standard deviations instead of their means. In A and B, the solid lines correspond to the case of no mismatch in the *a priori* inter-laminar current profiles. The dashed lines correspond to the case of a mismatch in the *a priori* inter-laminar current profiles (see text).

In this section we assess the performance of the inverse methods in the absence of errors in the *a priori* inter-laminar current profile. In other words, we assume that the current profiles are known *a priori*. The average reconstruction errors over 500 realizations are displayed in Fig 6 (solid lines). Before we consider performance differences between the methods, we have a look at the properties of the current reconstructions that are common to all methods. First, irrespective of the type of activation (local/global and superficial/deep), reconstruction errors increase with increasing noise-level. MNE and WMNE reconstruction errors for global activations seem to be an exception, but in this case, the average reconstruction errors are less accurate because of their large variance over realizations. Such a decrease in performance with increasing noise-level is to be expected because higher noise-levels lead to stronger regularization, which increases the bias in the reconstructions. Second, for noisy data, the errors vary more across measurements. This is because higher noise levels make it more difficult to select an appropriate value for the noise regularization parameter. This fact is relevant for experimental data, because in addition to yielding high errors *on average* (that is, over a large number of data-sets), high noise-levels make the reconstructions more uncertain. We also note that although the inverse methods considered in this study are linear, this is only true when the value of the noise regularization parameter is set. Indeed, when model tuning is viewed as part of the inverse method, the methods are non-linear. Third, deep generators are more difficult to reconstruct than superficial ones, especially for local activations. The reason for this is that the forward model is a spatial low-pass filter: spatial detail (short-wavelength activity) in the intra-laminar current profiles is suppressed and hence is harder to recover, especially in the presence of measurement noise. This is why deep generators are difficult to reconstruct especially for local activations.

Concerning the performance differences between the methods, we make two remarks. First, MNE and WMNE perform roughly equal and the same holds for LORETA and LORETA*. This means that weighting of the leadfields only has a small effect on the resulting reconstructions. To explain this, recall that for the low-resolution source space, each voxel that is covered by the electrode plane corresponds to an electrode: their are no voxels in-between electrodes (see Fig 1C). Therefore, the leadfields in the electrode plane have similar norms so that weighting by (a power of) their norms effectively doesn’t make a difference. For high-resolution imaging, leadfield weighting does make a difference (see Section **b1** of **Results**). Second, irrespective of the noise-level and generator depth; for local activations, the performance of MNE (and WMNE) and LORETA (LORETA*) is similar, while for global activations, LORETA performs much better than MNE. This is to be expected since global currents better agree with the *a priori* assumptions of LORETA (spatial smoothness). Corresponding results for unbalanced sources are shown in S2 Fig.

As described in Section **b1** of **Materials and Methods**, the different imaging methods require the specification of an *a priori* inter-laminar current profile. Since generally we cannot expect this profile to be accurately known, it is important to consider the methods’ performance in case of a mismatch between the true and *a priori* profiles. We conducted the same sets of simulations as above, but with the difference that in case of a superfical/deep generator, we *a priori* assume a deep/superficial generator (see Table 2). The results are shown in Fig 6 (dashed lines). We make several remarks. First, the mismatch affects LORETA/LORETA* differently than MNE/WMNE in that for LORETA/LORETA*, the changes in error means and standard deviations are independent of the noise-level, while for MNE/WMNE they depend on the noise-level. Second, for all methods, the increases in error means are larger for superficial than for deep generators. Third, while the mismatch lead to increases in the error variances for superficial generators, it generally leads to decreases for deep generators. The latter reflects a more stable selection of the regularization parameter λ. The cause of this is that if a deep generator is (erroneously) assumed to be superficial, its intra-laminar spatial extent will be overestimated, in order to fit the observed data. This is due to the fact that the LFP forward model acts like a spatial lowpass filter. Especially for local activations and noisy measurements, larger (assumed) sources are easier to distinguish from spatially uncorrelated noise, which is what (generalized) cross-validation tries to do when selecting an appropriate value for the regularization parameter. This also explains the increased error variance for superficial generators because (erroneously) assuming superfical generators to be deep, leads to underestimation of their intra-laminar spatial extent, which renders model tuning more difficult.

#### c2. The effect of electrode-montage

In the simulations above we have used the forward model given by Eq (12), which describes the relation between the CSD and the ensuing (trial-averaged) *absolute* electrical potential at the recording electrodes, that is, the potentials referenced to an electrode located at infinity. In practice, however, the reference electrode must be in contact with the preparation because electric potentials are measured indirectly via the current between the recording and reference electrode. For *in vivo* cortical LFPs, the reference electrode is often located at the surface of the contra-lateral cortex or on the skull. Because existing LFP volume-conductor models are local [9, 24] and therefore cannot simulate LFPs that are referenced to a distant electrode, the LFPs have to be *re-referenced*, prior to the estimation of the CSD. A common misconception in the field of EEG imaging is that switching to a different electrode montage (that is, re-referencing) does not influence the source reconstructions and this issue is given surprisingly little attention in the literature. In fact, most EEG (and ECoG) inverse modeling studies assess the performance of inverse methods on absolute potentials only [31] and whose practical relevance therefore, is limited. As far as we know, reference issues have not been discussed in the literature on LFP inverse modeling and existing studies have focused exclusively on absolute potentials [25–28, 30].

It is straightforward to show that re-referencing changes the LFP forward model and hence the CSD reconstructions. Consider again the horizontal forward model for absolute potentials (Eq (16)):

$$\begin{array}{c} V=G_{h}C_{h}+\xi , \end{array}$$ (30)

where $V\in R^{p\times 1}$, $C_{h}\in R^{n_{h}^{2}\times 1}$, and where $\xi \in R^{p\times 1}$ denotes Gaussian measurement noise with expectation zero and covariance matrix Σ_*ξ*_. Furthermore, let $M\in R^{q\times p}$ for certain *q* ≤ *p* denote a *montage transformation* that transforms the absolute potentials *V* to the re-referenced potentials *V*_*M*_:

$$\begin{array}{c} V^{M}=MV. \end{array}$$ (31)

The forward model for the re-referenced potentials is given by

$$\begin{array}{c} V^{M}=G_{h}^{M}C_{h}+\xi^{M}, \end{array}$$ (32)

where $G_{h}^{M}=MG\in R^{q\times n_{h}^{2}}$ denotes the re-referenced horizonal leadfield matrix and

$$\begin{array}{c} \xi^{M}=M\xi , \end{array}$$ (33)

denotes the re-referenced measurement noise, which has covariance matrix *M*Σ_*ξ*_ *M*^*t*^. The re-referenced horizontal forward model shows that re-referencing has two effects: It changes the leadfield matrix and it changes the covariance structure of the measurement noise. Because we have assumed the data to be prewhitened, *MΣ*_*ξ*_ *M*^*t*^ is a scaled copy of the identity matrix. Moreover, since we will focus on the *average-reference montage*, which corresponds to taking $M=1_{p}-e_{p}e_{p}^{t}$, where $e_{p}\in R^{p\times 1}$ denotes the vector containing all ones, *MM*^*t*^ is close to the identity matrix and hence *M*Σ_*ξ*_ *M*^*t*^ approximately equals Σ_*ξ*_. We therefore focus on the effect that re-referencing has on the leadfield matrix.

**Fig 7:**
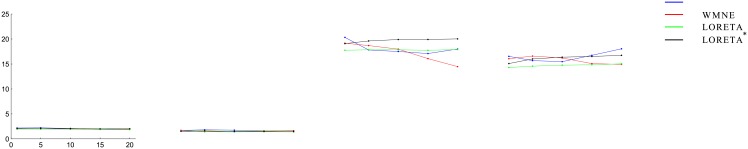
Effects of re-referencing the data. Differences in mean reconstruction errors obtained using the average-reference montage and the single-wire montage for the four inverse methods (MNE (blue), WMNE (red), LORETA (green), and LORETA* (black)) as a function of noise-level and for each of the four combinations of simulated currents (superficial/deep and local/global). Noise-levels are 1, 5, 10, 15, and 20%. The mean errors were obtained by averaging over 500 realizations.

To assess the effect of re-referencing on the quality of the CSD reconstructions, we carried out the standard set of simulations, but used average-referenced potentials instead of absolute potentials. Fig 7 shows the difference in mean reconstruction errors with those obtained by using single-wire potentials. Note that for local activations, changing from absolute potential to average-reference potentials has practically no effect on the mean reconstruction errors and this holds for all methods. In contrast, for global activations, changing to the average-reference montage drastically increased the mean errors. Also note that these effects are independent of the noise-level. It turns out that the large errors for global activations can largely be accounted for by errors in the off-set of the reconstructions. This we checked by recalculating the errors modulo an additive constant: the resulting errors were practically the same as those obtained from the absolute potentials. The errors in off-set arise because imaging average-reference potentials yields approximately balanced (that is, sum-zero) intra-laminar current profiles. Since localized activations are approximately balanced, error don’t increase much. Global activations, however, tend to be unbalanced (see Fig 2B), giving rise to larger errors when using the average-reference montage.

### D. Evoked responses in macaque primary visual cortex

**Fig 8:**
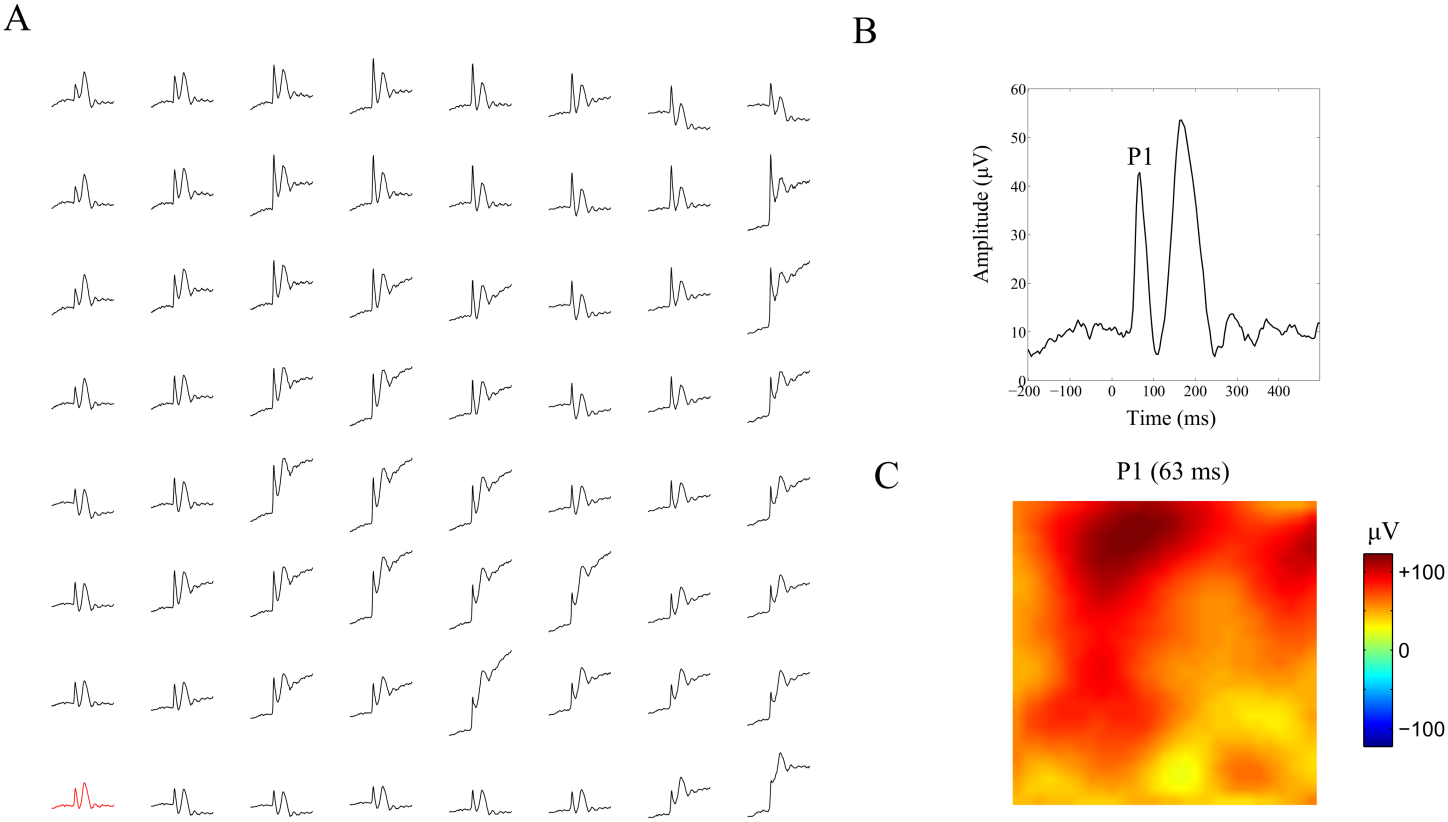
Evoked potentials in macaque primary visual cortex. A. Multiplot of the visually evoked potentials (VEPs) recorded at the 64 electrodes of the Utah array. Time ranges from 200 ms pre-stimulus to 500 ms post-stimulus. B. Close-up of the VEP at the lower-left corner of the electrode array (red trace in A). Time is relative to stimulus onset. The peaks selected for analysis is indicated by P1 and has a latency of 63 ms. C. Topographic map of P1. All potentials are relative to the single-wire reference.

In this section we apply inverse modeling to reconstruct the intra-laminar CSD underlying visually evoked potentials (VEPs) in macaque primary visual cortex (V1) recorded with an 8 × 8 intracortical Utah array with 400 *μ*m inter-electrode spacing. Fig 8A shows a multiplot of the VEPs where each trace corresponds to a recording electrode. Fig 8B shows a close-up of the VEP recorded at the electrode in the lower-left corner of the array (red trace in A). We focus on inverse modeling of the early positive peak (P1) at a latency of 63 ms, which is known to reflect direct input from the thalamus into cortical layer 4 and can be modeled using a single dipolar generator [14]. Fig 8C shows the single-wire potential topography of P1. Observe that the map has a single sign, which likely reflects (stimulus-locked) neural responses in the vicinity of the reference electrode. Before inverting P1, we therefore transformed it to the average-reference montage. In Section **c2** of **Results**, we have established that the average-reference potentials only allow reconstruction of relative CSDs and that the reconstruction errors are similar to those obtained using single-wire potentials. The source space was chosen identical to that used in the simulations and the leadfield matrix was recalculated for the (8 × 8)) electrode layout. Furthermore, since the conduction properties of the modeled tissue have only a modest influence on the CSD reconstructions, we assumed isotropy and homogeneity.

**Fig 9:**
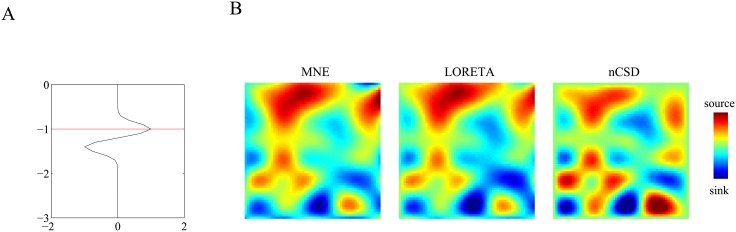
P1 reconstructions. A. *A priori* inter-laminar CSD profile using in the inversion of P1. The profile is modeled by a dipolar generator of length 0.4 mm and is located 1.2 mm below the (modeled) pial surface. The horizontal red line indicates the depth of electrode plane. B. Reconstructed CSDs underlying P1 obtained using MNE (left), LORETA (middle), and the (numerical) CSD method (right). Blue and red correspond to superficial and deep generators, respectively.

We inverted P1 using an *a priori* inter-laminar profile comprising a single dipolar generator located 2 mm below the recording array (see Fig 9A) and inverted P1 using MNE, LORETA, and the CSD method. The reconstructions obtained using MNE and LORETA were tuned using generalized cross-validation (GCV). The reconstructions are shown in Fig 9B. We make several remarks. First, the MNE and LORETA reconstructions are relatively similar, which is an indication that the true CSD is local, that is, contains power at high spatial frequencies. Second, the nCSD reconstruction is different from the MNE and LORETA reconstructions. In particular, it shows local sources and sinks that are suppressed in the MNE and LORETA reconstructions. It is important to notice that these local currents follow the layout of the electrode array because this indicates that the true CSD is undersampled. In other words, the typical length-scale over which the true CSD is coherent, most likely is lower than 400 *μm* (which is the inter-electrode distance). The reason why the inverse reconstructions (MNE and LORETA) are less affected by this is that the prior inter-laminar CSD profile is chosen close to the array: when placed deeper, the reconstructions are more affected and become similar to the nCSD reconstruction.

## Discussion

In this study we have described a general way to construct discrete volume-conduction models for LFP recordings that avoids the singularities that arise when using point-sources and allows the simulation of arbitrary three-dimensional current-source densities (CSDs). We have constructed such a volume-conduction model for cortical Utah array recordings and used it to investigate the applicability of the most commonly used linear distributed inverse methods in the field of EEG/MEG imaging [31] to planar LFP data. We also have illustrated these methods on early evoked potentials in macaque primary visual cortex. Our overall conclusion is that such methods can indeed be applied to planar LFP recordings and that they have the potential to yield more accurate CSD reconstructions than the classical (planar) CSD method. To what extent they outperform the CSD method, however, depends on a number of factors, most notably the accuracy of the *a priori* inter-laminar CSD profile. Our study raised a number of issues, however, that warrant more discussion.

Concerning the factors that affect the performance of the different inverse methods, our simulations suggest that the most important factor is the *a priori* inter-laminar CSD profile. The exact values for tissue anisotropy and homogeneity are much less crucial. This implies that for successful application of linear distributed inverse methods to planar LFP recordings, the inter-laminar CSD profile has to be known rather accurately, which can only be obtained by (not necessarily simultaneous) inter-laminar recordings from the same preparation. In exchange for this practical inconvenience, however, are possibly high-accuracy reconstructions of the current sources and sinks underlying the recorded field potentials. We also note that even the most simple and unrealistic *a priori* inter-laminar current profile (constant and finite) is more realistic than the profile presupposed by the two-dimensional CSD method (constant and infinite). Thus, in the absence of *a priori* knowledge about the electrical properties of the tissue at hand and the inter-laminar organization of the currents, LFP inverse methods force us to make explicit our assumptions regarding the preparation and allow us to explore the possible current profiles by varying model parameters. These reasons already show why using forward modeling in the analysis of planar LFP recordings is beneficial.

An interesting (and somewhat unexpected) finding is that the Utah array seems to undersample the intra-laminar current profile of the early visually evoked response at least at some locations. This is manifested by spurious sources and sinks in the reconstructed current profiles that have the dimensions of the distance between the recording electrodes (Fig 9B). We have simulated intra-laminar CSD profiles comprising generators whose characteristic scale < 400 *μ*m (the inter-electrode distance) which yield the same spurious sources and sinks. Generally, spatial aliasing (undersampling) occurs when the inter-electrode distance is larger than half the radius of the sources and sinks (Nyquist sampling theorem). The presence of spurious sources and sinks in our reconstructions therefore implies that the radii of the true sources and sinks < 800 *μ*m and, based on our simulations, most likely < 400 *μ*m. The reason why the inverse reconstructions are less affected by spatial aliasing then the CSD method is that by using an explicit forward model, they take into account the spatial low-pass effects of volume-conduction. Indeed, anti-aliasing filters act as lowpass spatial filters and have been applied to CSD reconstructions (obtained by the CSD method) from planar CSD recordings [19]. Even with the use of an explicit forward model, however, spurious sources and sinks still seem to be present (Fig 9B) which indicates that the sampling resolution of the electrode array is just not sufficient to recover all high spatial frequencies present in the true intra-laminar CSD profile.

The source-model used in this study is limited in several ways. First, we have only considered CSDs with balanced (that is, sum-zero) inter-laminar profiles, while recently, unbalanced cortical CSDs have been reported [23]. An unbalance in the inter-laminar current profile, however, can easily be incorporated in our source-model (Eq (28)) by separately weighting the amplitudes of the generator poles. In SF2, we show the reconstruction errors in the case of a particular unbalance in the inter-laminar profile. Fig 6 shows that the resulting errors are larger than the errors for the corresponding balanced profiles. These preliminary simulations show that mono-polar and higher-order >2) terms in the multi-pole expansion of the extra-cellular potential, do influence the performance of imaging methods, in line with [29]. Second, following earlier inverse modeling studies of planar LFPs [26, 27], the CSD is assumed to be a product of real-valued inter- and intra-laminar profiles. The fact that the profiles are assumed to be real-valued means that intra- and inter-laminar phase-differences cannot be modeled. Incorporating intra-laminar phase differences is important when inverting spontaneous cortical oscillations, which often exhibit phase-differences between lamina [1, 3, 4, 16, 45, 50]. Incorporating inter-laminar phase-differences enables more accurate inversion of event-related activity, whose laminar organization can be heterogeneous over the tissue covered by the recording array. Although intra- and inter-laminar phase-differences can be incorporated into the estimation framework by extending the source model to the complex domain, it is unclear under which conditions and assumptions the phase-profiles can be accurately reconstructed. Third, the intra-laminar CSD profile is assumed to be known, which is not always the case in experimental applications. When the intra-laminar CSD profile is not fixed *a priori*, minimum norm estimators will concentrate the estimated CSD around the electrode (proximity bias in the inter-laminar direction). A possible approach to reconstruct the inter-laminar organization directly from the LFPs would be adaptive spatial filtering [51, 52], which is a popular approach to invert EEG/MEG data [53]. Additional advantages of spatial filters over the minimum-norm type inverse methods treated in our study is that they do not suffer from surface bias, are more robust to interfering sources, and might allow reconstruction of currents that are not directly located underneath the electrode array. A major disadvantage is their disability to deal with correlated activity and therefore need to be adapted in some way to be applicable to LFP recordings.

Our study entirely focused on the performance of several linear distributed inverse methods from the field of EEG/MEG inverse imaging and we did not carry out a comparison with existing inverse methods for planar LFPs [26, 27]. We can make a number of remarks, however. With respect to the inverse current source density (iCSD) method proposed in [26], we note that it is a special case of linear distributed inverse methods. Recall that linear distributed inverse methods stabilize the inversion of the leadfield matrix by adding a regularization term. Alternatively, when using the low-resolution source space, the leadfields can be restricted to those that correspond to the electrodes, yielding a square matrix that is inverted directly (if it has full rank), which is what iCSD does. Thus, iCSD is obtained by restricting the low-resolution source space and setting the noise regularization parameter to zero. Note that the restriction of the leadfields corresponds to the assumption that all currents are confined to the electrode grid. Other drawbacks of the iCSD method are that it cannot be generalized to high-resolution source spaces and that it is sensitive to measurement noise (because of the absence of a regularization term). An interesting direction for future research will be to provide a general statistical framework containing all LFP inverse methods. Such a framework yields conceptual clarity and will enable to directly compare the performance of the different inverse methods.

## Supporting information

S1 Text Derivation of the extra-cellular potential of a charged cube within an infinite anisotropic volume conductor. (PDF)

S1 Fig Reconstruction of distant sources. A. Reconstruction errors for all four imaging methods (MNE, WMNE, LORETA, and LORETA*) as a function of the *y*-location of a single two-dimensional Gaussian source with *x*-coordinate at the center of the grid. The black vertical bars denote the *y*-coordinates of the electrodes at the boundary of the grid. The errors were calculated by taking into account the entire source-space (thus no restriction to the electrodes as in the main text). The *y*-coordinate ranged through the (low-resolution) source space in steps of 0.1 mm. The intra-laminar width of the source was set to 0.5 mm. Measurement noise was absent. The source was located at a depth of 1.4 mm. B. True (first panel) and reconstructed (second to fifth panel) intra-laminar CSD. In this case, the (center of the) source was located 0.8 to the left of the grid boundary.
(EPS)

S2 Fig Performance of the imaging methods for unbalanced sources. Mean reconstruction errors for the four inverse methods (MNE (blue), WMNE (red), LORETA (green), and LORETA* (black)) as a function of noise-level and for each of the four combinations of simulated currents (superficial/deep and local/global). Noise-levels are 1, 5, 10, 15, and 20%. The mean errors were obtained by averaging over 500 realizations. To unbalance the inter-laminar current profiles, the amplitude of the lower generator pole was set to half its value.
(EPS)
