# Supplementary material for: Linear distributed source modeling of local field potentials recorded with intra-cortical electrode arrays
Source: PLoS One. 2017 Dec 18;12(12):e0187490. doi: 10.1371/journal.pone.0187490 (PMC5734682; doi:10.1371/journal.pone.0187490)
Supplement: S1 Text — (PDF) [file pone.0187490.s001.pdf]

The potential of a homogeneous voxel

Below, we generalize the formula derived in (Hummer, 1996) to the case of anisotropic voxels and simplify the expressions. Let the voxel be given by the rectangular volume $[-a,a]\times[-b,b]\times[-c,c]$ for certain constants $a,b,c>0$ and suppose that $\rho=1$ on the rectangle. The electric potential is then is given by

$$V(u,v,w)=\frac{1}{4\pi}\int_{-a}^{a}\int_{-b}^{b}\int_{-c}^{c}\frac{dxdydz}{\sqrt{\sigma_y\sigma_z(x-u)^2+\sigma_z\sigma_x(y-v)^2+\sigma_x\sigma_y(z-w)^2}} \tag{1}$$

Integrating by parts yields

$$\begin{aligned}2V(u,v,w) &= \frac{1}{4\pi}\int_{-a-u}^{a-u}\int_{-b-v}^{b-v}\frac{zdxdy}{\sqrt{\sigma_u^2x^2+\sigma_v^2y^2+\sigma_w^2z^2}}\Big|_{z_0}^{z_1}\\ &+ \frac{1}{4\pi}\int_{-a-u}^{a-u}\int_{-c-w}^{c-w}\frac{ydxdz}{\sqrt{\sigma_u^2x^2+\sigma_v^2y^2+\sigma_w^2z^2}}\Big|_{y_0}^{y_1}\\ &+ \frac{1}{4\pi}\int_{-b-v}^{b-v}\int_{-c-w}^{c-w}\frac{xdydz}{\sqrt{\sigma_u^2x^2+\sigma_v^2y^2+\sigma_w^2z^2}}\Big|_{x_0}^{x_1},\end{aligned}$$

where we have defined $\sigma_u=\sqrt{\sigma_y\sigma_z}$, $\sigma_v=\sqrt{\sigma_z\sigma_x}$ and $\sigma_w=\sqrt{\sigma_x\sigma_y}$. By using the identities

$$\int_{y_0}^{y_1}\frac{dy}{\sqrt{x^2+y^2+z^2}}=\log\frac{\sqrt{x^2+y_1^2+z^2}+y_1}{\sqrt{x^2+y_0^2+z^2}+y_0},$$

and

$$\begin{aligned}\int_{x_0}^{x_1}\log\left(\sqrt{x^2+y^2+z^2}+y\right)dx &= y\log\left(\sqrt{x^2+y^2+z^2}+x\right)\\ &+ x\log\left(\sqrt{x^2+y^2+z^2}+y\right)\\ &- z\arctan\left(\frac{xy}{z\sqrt{x^2+y^2+z^2}}\right)\\ &- x+z\arctan\left(\frac{x}{z}\right),\end{aligned}$$

we organize the solution to by defining functions f, g and h as follows:

$$\begin{aligned}f(x,y,z) &= yz\log\left(\sqrt{x^2+y^2+z^2}+x\right)+xz\log\left(\sqrt{x^2+y^2+z^2}+y\right)-\\ &\quad z^2\arctan\left(\frac{xy}{z\sqrt{x^2+y^2+z^2}}\right),\end{aligned}$$

which is well-defined for all x,y,z, including at the origin where the argument of the arctan function above assumes the limiting value $1/\sqrt{3}$,

$$\begin{aligned}g(x_1,x_0,y_1,y_0,z_1,z_0) &= [\{f(x_1,y_1,z_1)-f(x_0,y_1,z_1)\}-\{f(x_1,y_0,z_1)-f(x_0,y_0,z_1)\}]-\\ &\quad [\{f(x_1,y_1,z_0)-f(x_0,y_1,z_0)\}-\{f(x_1,y_0,z_0)-f(x_0,y_0,z_0)\}],\end{aligned}$$

and

$$\begin{aligned}h(a,b,c,u,v,w) &= g(a-u,-a-u,b-v,-b-v,c-w,-c-w)+\\ &\quad g(b-v,-b-v,c-w,-c-w,a-u,-a-u)+\\ &\quad g(c-w,-c-w,a-u,-a-u,b-v,-b-v).\end{aligned}$$

In terms of these functions, and in the case of $\sigma_x = \sigma_y = \sigma_z = 1$, the potential is given by

$$V(u, v, w) = \frac{1}{4\pi} \frac{h\left(a, b, c, u, v, w\right)}{2}$$

The general solution to follows by a shift in the arguments of h: $a \to \sigma_u a$, $b \to \sigma_v b$, $c \to \sigma_w c$, $u \to \sigma_u u$, $v \to \sigma_v v$, $w \to \sigma_w w$ and a multiplicative factor:

$$V(u, v, w) = \frac{1}{4\pi} \frac{h\left(\sigma_u a,\, \sigma_v b,\, \sigma_w c,\, \sigma_u u,\, \sigma_v v,\, \sigma_w w\right)}{2\sigma_u \sigma_v \sigma_w}. \tag{2}$$
